# Supplementary material for: Single-molecule imaging reveals a direct role of CTCF’s zinc fingers in SA interaction and cluster-dependent RNA recruitment
Source: Nucleic Acids Res. 2024 May 14;52(11):6490–506. doi: 10.1093/nar/gkae391 (PMC11194110; doi:10.1093/nar/gkae391)
Supplement: gkae391_Supplemental_File [file gkae391_supplemental_file.pdf]

Supplementary Information

Single-molecule imaging reveals a direct role of CTCF's zinc fingers in SA interaction and cluster-dependent RNA recruitment

Jonas Huber[1], Nicoleta-Loredana Tanasie[1], Sarah Zernia[1], Johannes Stigler[1,*]

[1] Gene Center Munich, Ludwig-Maximilians-Universität München, Munich, Germany
* To whom correspondence should be addressed: stigler@genzentrum.lmu.de

<u>This file contains:</u>

Supplementary figures

Significance tests

Protein sequences

Supplementary figures

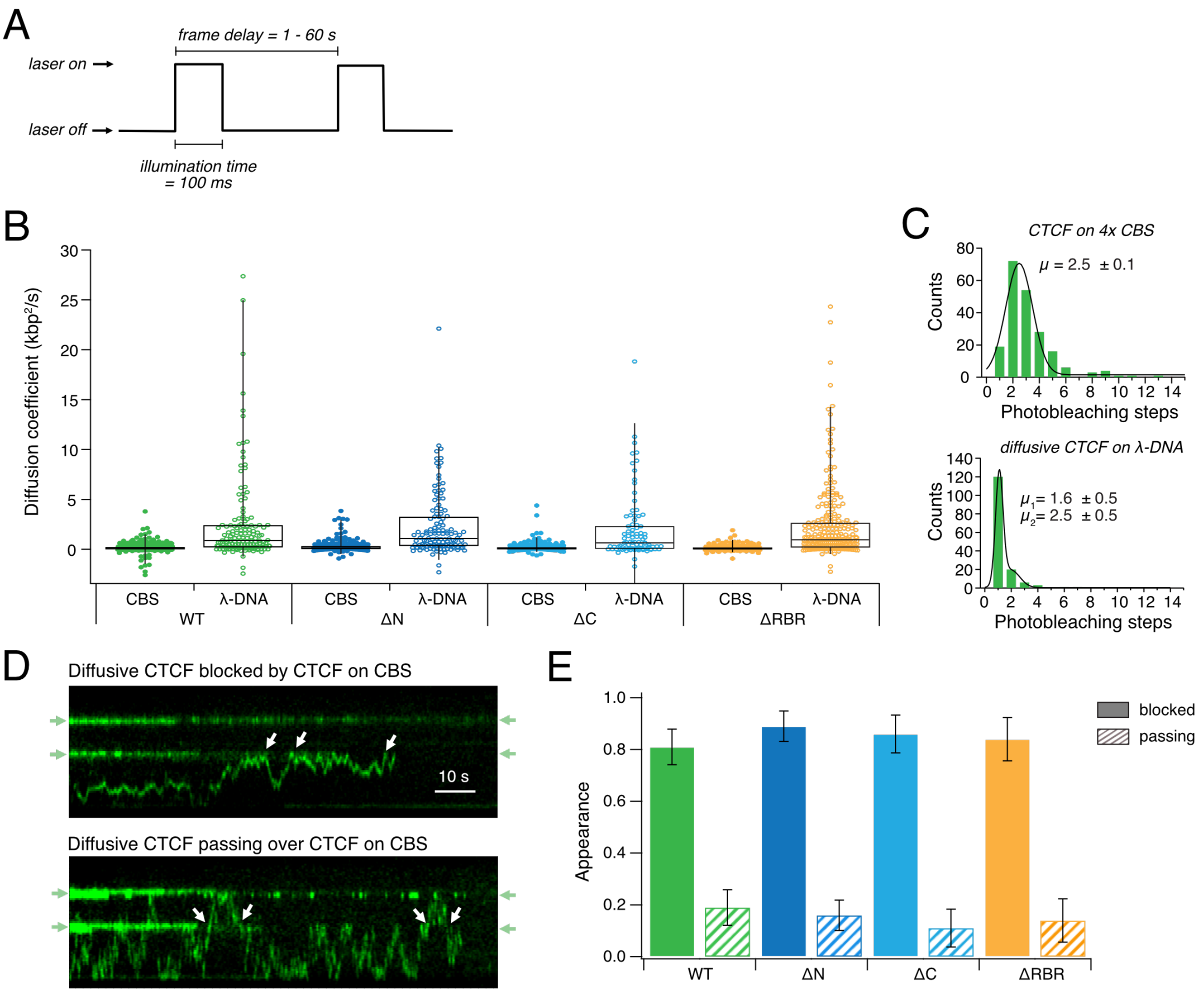

Figure S1. CTCF diffuses on non-CBS sites. **(A)** Scheme illustrating laser illumination during lifetime measurements. **(B)** Diffusion coefficients of AF568-CTCF WT and variants on 4x CBSs or λ-DNA at 10 nM concentration and 100 ms illumination time. For all variants, D is significantly higher on λ-DNA than CBSs. No significant difference in diffusive behavior between CTCF variants. **(C)** Photobleaching steps of non-diffusive (top) and diffusive (bottom) CTCF. **(D)** Representative kymograms showing diffusion behavior of WT CTCF. Top: White arrows indicate events where diffusive CTCF is blocked by CBS-bound CTCF. No recruitment of diffusive CTCF to the binding sites occurs. Bottom: White arrows indicate events where diffusive CTCF passes CBS-bound CTCF. **(E)** Quantification of blocking and passing events. No significant differences were observed between WT and CTCF variants.

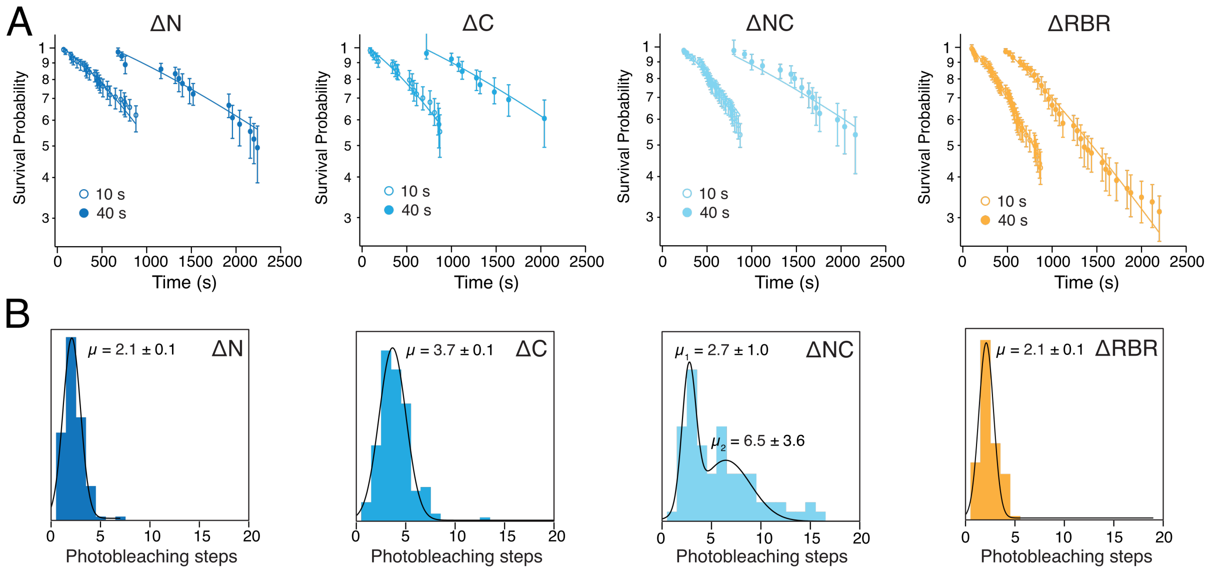

Figure S2. Lifetimes of AF568-CTCF variants at 10 nM concentration. **(A)** Lifetimes of CTCF variants at 10 and 40 s frame delay and 100 ms illumination time. **(B)** Photobleaching steps of CTCF variants binding to 4x CBSs. Black line: Multi-Gaussian fit.

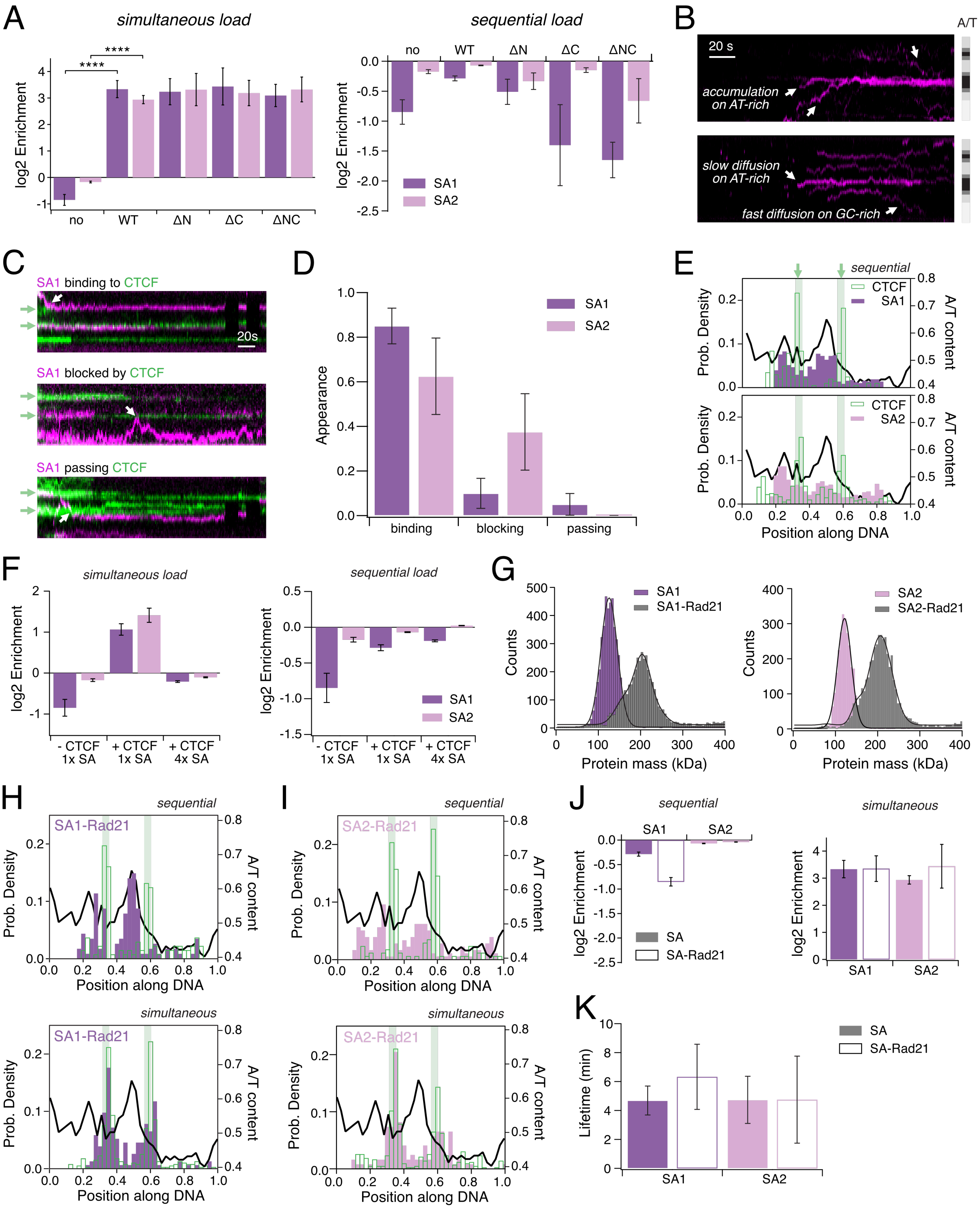

Figure S3. SA diffusion behavior and influence of Rad21 on CTCF-SA interaction. **(A)** Enrichment of SA1 (purple) and SA2 (pink) on CBSs. During simultaneous load (left), both SA-LD655s are enriched significantly more on CBSs when preincubated at 100 nM concentration with 10 nM AF568-CTCF WT compared to SAs loaded alone. SA is enriched similarly in presence of all CTCF variants. When 10 nM AF568-CTCF was bound first to DNA, followed by salt enrichment and by 100 nM SA-LD655 (sequential load, right), no significant SA enrichment at CBSs was observed with any CTCF variant. **(B)** Representative kymograms of SAs binding static to AT-rich and diffusing randomly on GC-rich

regions. **(C)** Representative kymograms of binding (top), blocking (middle) and passing (bottom) events observed for diffusive SAs (magenta) after sequential load on CTCF (green). **(D)** Fraction of binding, blocking and passing events observed for SA1 and SA2 after sequential load. No significant difference was found between SA1 and SA2. **(E)** Histograms of CTCF (green, top: N = 203; bottom: N = 410), SA1 (N = 580) and SA2 (N = 519) binding positions after sequential load at 4 x SA concentration (10 nM AF568-CTCF followed by 300 mM NaCl enrichment and 400 nM SA-LD655). CBSs are shown as green bars and the AT-ratio as a black line. **(F)** Left: enrichment of SAs on CBSs in absence of CTCF and at 1 x or 4 x SA concentrations, simultaneous load with CTCF, without salt enrichment. Right: enrichment of SAs on CBSs in absence of CTCF and at 1x or 4 x SA concentrations, sequential load after CTCF salt enrichment. **(G)** Mass photometry data of 148 kDa SA1 and 145 kDa SA2 in absence and presence (gray) of 59 kDa Rad21-MBP. **(H)** Histogram of SA1-Rad21 and CTCF binding positions for sequential load (top, CTCF, N = 137; SA1-Rad21, N = 114) and simultaneous load (bottom, CTCF, N = 397; SA1-Rad21, N = 153) experiments. **(I)** Same as (E) but using SA2-Rad21 (sequential, CTCF, N = 292; SA2-Rad21, N = 257; simultaneous, CTCF, N = 433; SA2-Rad21, N = 122). **(J)** Enrichment of SAs on CBSs in absence (solid) or presence (transparent) of Rad21 for sequential (left) and simultaneous load (right). Enrichments are independent from Rad21. **(K)** Lifetimes of SAs on CTCF in absence (solid) and presence (transparent) of Rad21. Lifetimes are independent of Rad21. All experiments were carried out at 100 ms illumination time.

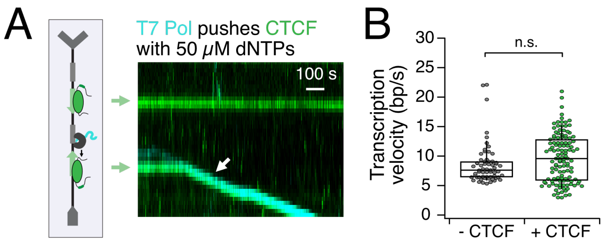

Figure S4. In vitro transcription assay at low nucleotide concentration **(A)** Illustration of in-vitro transcription assays and representative kymogram for T7-Pol pushing CTCF off its site during transcription at 50 μM nucleotide concentration (cyan = Cy3-UTP labeled RNA, green = AF568- CTCF) **(B)** Mean transcription velocities of T7-Pol alone and T7-Pol pushing CTCF WT at 50 μM nucleotide concentration.

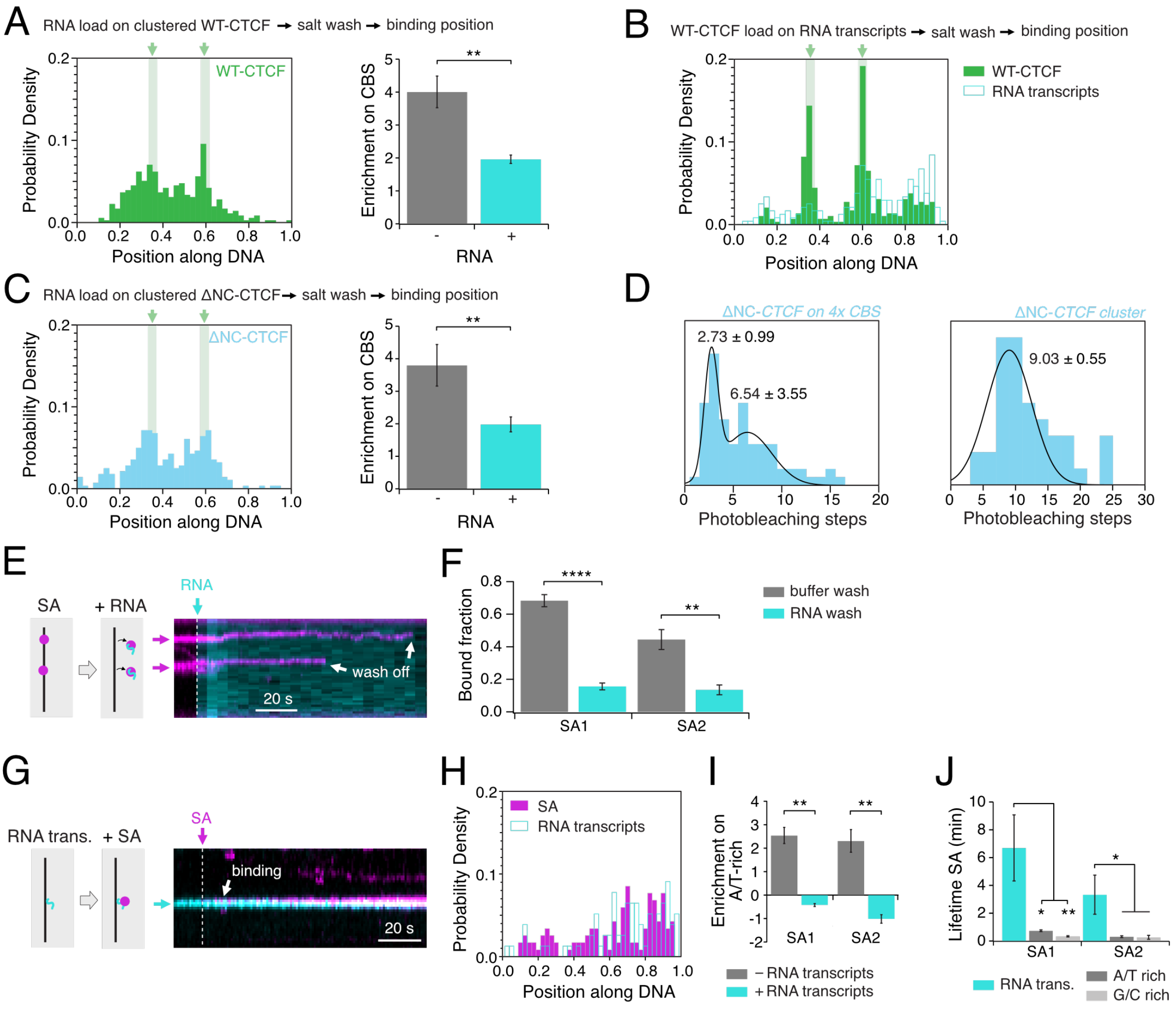

Figure S5. SA has a higher affinity for RNA than for DNA. **(A)** Left: Histogram of AF568-CTCF-WT-RNA cluster (N = 355) binding positions after salt enrichment (see Figure 5D). Right: CTCF WT-RNA clusters are significantly less enriched on CBSs than monomeric CTCF WT. **(B)** Histogram of CTCF WT binding positions after transcription and salt enrichment (see Figure 5G). CTCF preferentially binds to 4x CBSs but also colocalizes with RNA transcripts (CTCF, N = 292; RNA, N = 237). **(C)** Left: Histogram of AF568-CTCF-ΔNC-RNA cluster (N = 280) binding positions after salt enrichment. Right: ΔNC-RNA clusters are significantly less enriched on CBSs than monomeric ΔNC. **(D)** Histogram of photobleaching steps for ΔNC on 4x CBSs (same as Figure S2B-3) and in RNA-clusters. **(E)** Scheme and representative kymogram of SAs being washed off from λ-DNA by the addition of RNA to the DNA-curtain. **(F)** More 100 nM SA-LD655 is washed off in the presence of 25 ng/μl Cy3-UTP labeled RNA than in the presence of buffer. **(G)** Scheme and representative kymogram of SAs colocalizing with RNA transcripts. **(H)** Histogram of SA1 binding positions after transcription (SA1, N = 43; RNA, N = 155). **(I)** SA1 and SA2 are significantly less enriched on AT-rich regions in presence of RNA transcripts on the DNA. **(J)** SA1 and SA2 have a significantly higher lifetime on RNA transcripts than on AT-rich or GC-rich DNA regions. All experiments were carried out at 100 ms illumination time.

Significance tests

	p-values	N
**Figure 1**		
(F) 4x CBSs/1x CBS	0.016 (t)	427/477
(H) 4x CBSs/1x CBS	0.89 (z)	701/201
(H) 4x CBSs/λ-DNA	$< 10^{-6}$ (z)	701/271
(H) 1x CBS/λ-DNA	$< 10^{-6}$ (z)	201/271
**Figure S1**		
(B) λ-DNA /CBS WT	$2.6 * 10^{-6}$ (t)	148/261
(B) λ-DNA /CBS ΔN	$< 10^{-6}$ (t)	125/235
(B) λ-DNA /CBS ΔC	$1.4 * 10^{-6}$ (t)	89/288
(B) λ-DNA / CBS ΔRBR	$< 10^{-6}$ (t)	266/189
(B) WT/ΔN λ-DNA	0.37 (t)	148/125
(B) WT/ΔC λ-DNA	0.19 (t)	148/89
(B) WT/ΔRBR λ-DNA	0.24 (t)	148/266
(E) WT/ΔN	0.48 (f)	32/28
(E) WT/ΔC	0.72 (f)	32/22
(E) WT/ΔRBR	1 (f)	32/19
**Figure 2**		
(B) WT/ΔN	0.22 (t)	427/390
(B) WT/ΔC	0.63 (t)	427/666
(B) WT/ΔNC	0.49 (t)	427/396
(B) WT/ΔRBR	0.26 (t)	427/651
(B) WT/ZF9-CT	0.0012 (t)	427/343
(B) WT/ZF4-7	0.0020 (t)	427/88
(D) WT/ΔN	0.97 (z)	701/125
(D) WT/ΔC	0.93 (z)	701/80
(D) WT/ΔNC	0.89 (z)	701/161
(D) WT/ΔRBR	$3.4 * 10^{-6}$ (z)	701/217
(D) WT/ZF9-CT	$< 10^{-6}$ (z)	701/155
(D) WT/ZF4-7	$< 10^{-6}$ (z)	701/88
**Figure 3**		
(G) 50 mM NaCl SA1/SA2 GC	0.012 (z)	186/48
(G) 50 mM NaCl SA1/SA2 AT	0.37 (z)	54/62
(G) 50 mM NaCl GC/AT SA1	$< 10^{-6}$ (z)	54/186
(G) 50 mM NaCl GC/AT SA2	0.0019 (z)	62/48
(G) 150 mM NaCl SA1/SA2 GC	0.63 (z)	8/20
(G) 150 mM NaCl SA1/SA2 AT	$1.2 * 10^{-6}$ (z)	65/62
(G) 150 mM NaCl GC/AT SA1	$< 10^{-6}$ (z)	8/65
(G) 150 mM NaCl GC/AT SA2	0.77 (z)	20/62
(H) SA1/SA2 GC	0.0058 (z)	537/510
(H) SA1/SA2 AT	0.92 (z)	231/169
(H) GC/AT SA1	$2.1 * 10^{-5}$ (z)	537/231
(H) GC/AT SA2	$< 10^{-6}$ (z)	510/169

(I) WT/DNA SA1	7.7 * 10^{-5} (z)	65/8
(I) WT/DNA SA2	0.0044 (z)	62/20
(I) WT/ ΔN SA1	0.41 (z)	65/28
(I) WT/ΔC SA1	0.62 (z)	65/20
(I) WT/ΔNC SA1	0.76 (z)	65/9
(I) WT/ΔN SA2	0.83 (z)	62/28
(I) WT/ΔC SA2	0.66 (z)	62/17
(I) WT/ΔNC SA2	0.85 (z)	62/10
**Figure S3**		
(A)WT/DNA SA1 simultaneous	2.5 * 10^{-4} (t)	332/1321
(A)WT/DNA SA2 simultaneous	3.3 * 10^{-6} (t)	224/875
(A)WT/ΔN SA1 simultaneous	0.74 (t)	332/197
(A)WT/ΔN SA2 simultaneous	0.31 (t)	224/153
(A)WT/ΔC SA1 simultaneous	0.79 (t)	332/251
(A)WT/ΔC SA2 simultaneous	0.41 (t)	224/205
(A)WT/ΔNC SA1 simultaneous	0.39 (t)	332/116
(A)WT/ΔNC SA2 simultaneous	0.22 (t)	224/40
(A)WT/DNA SA1 sequential	0.22 (t)	380/1321
(A)WT/DNA SA2 sequential	0.76 (t)	80/875
(A)WT/ΔN SA1 sequential	0.73 (t)	380/114
(A)WT/ΔN SA2 sequential	0.85 (t)	80/88
(A)WT/ΔC SA1 sequential	0.55 (t)	380/265
(A)WT/ΔC SA2 sequential	0.97 (t)	80/242
(A)WT/ΔNC SA1 sequential	0.18 (t)	380/207
(A)WT/ΔNC SA2 sequential	0.63 (t)	80/95
(D)SA1/SA2 binding	0.31 (f)	20/8
(D)SA1/SA2 blocking	0.12 (f)	20/8
(D)SA1/SA2 passing	1.0 (f)	20/8
(F)WT/DNA 1 x SA1 simultaneous	0.029 (t)	531/1321
(F)WT/DNA 1 x SA2 simultaneous	0.029 (t)	177/875
(F)WT/DNA 4 x SA1 simultaneous	0.13 (t)	418/1321
(F)WT/DNA 4 x SA2 simultaneous	0.86 (t)	1156/875
(F)WT/DNA SA1 sequential	0.22 (t)	380/1321
(F)WT/DNA SA2 sequential	0.76 (t)	80/875
(F)WT/DNA 4 x SA1 sequential	0.085 (t)	580/1321
(F)WT/DNA 4 x SA2 sequential	0.59 (t)	519/875
(J)SA1/SA1Rad21 on WT simultaneous	0.95 (t)	332/137
(J)SA2/SA2Rad21 on WT simultaneous	0.31 (t)	224/277
(J)SA1/SA1Rad21 on WT sequential	0.15 (t)	380/153
(J)SA2/SA2Rad21 on WT sequential	0.90 (t)	80/122
(K)SA1/SA1Rad21 on WT	0.51 (z)	65/16
(K)SA2/SA2Rad21 on WT	1.0 (z)	62/9
**Figure 4**		
(A)CTCF/no	0.78 (t)	3/3
(B)CTCF/no	0.18 (t)	3/3

(F)T7/WT	0.034 (t)	111/88
(F)T7/ΔN	0.22 (t)	111/84
(F)T7/ΔC	0.89 (t)	111/76
(F)T7/WT-SA1	0.63 (t)	111/9
(F)T7/WT-SA2	0.85 (t)	111/9
(H)T7single/T7multiple	$< 10^{-6}$ (f)	49/118
(H)T7single/WTsingle	$< 10^{-6}$ (f)	49/40
(H)T7single/WTmultiple	$< 10^{-6}$ (f)	49/147
(H)T7single/ΔNsingle	$4.1 * 10^{-6}$ (f)	49/33
(H)T7single/ΔNmultiple	$< 10^{-6}$ (f)	49/134
(H)T7single/ΔCsingle	$< 10^{-6}$ (f)	49/34
(H)T7single/ΔCmultiple	$< 10^{-6}$ (f)	49/177
(H)T7multiple/WTmultiple	0.0056 (f)	118/147
(H)T7multiple/ΔNmultiple	0.0031 (f)	118/134
(H)T7multiple/ΔCmultiple	0.042 (f)	118/177
**Figure S4**		
(B) T7/WT	0.075 (t)	61/121
**Figure 5**		
(H)RNA/noRNA enrichment 4x CBSs	0.060 (t)	292/427
(I) RNA/4x CBSs	$< 10^{-6}$ (z)	118/201
(I) RNA/λ-DNA	0.11 (z)	118/274
**Figure S5**		
(A)RNAcluster/monomers WT	0.0021 (t)	355/427
(C)RNAcluster/monomers ΔNC	0.0022 (t)	280/396
(F)RNA/noRNA wash off SA1	$< 10^{-6}$ (t)	560/479
(F)RNA/noRNA wash off SA2	0.00267 (t)	148/198
(I)RNA/noRNA AT-enrichment SA1	0.0029 (t)	1321/43
(I)RNA/noRNA AT-enrichment SA2	0.0088 (t)	875/117
(J)RNA/AT	0.012 (z)	420/260
(J)RNA/AT	0.032 (z)	295/119
(J)RNA/GC	0.0075 (z)	420/93
(J)RNA/GC	0.030 (z)	295/146

Table S1. Number of molecules and p-values for all relevant experiments. Two tailed t-test: (t), Two tailed z-test: (z), Fisher's exact test: (f).

Protein Sequences

CTCF WT
6xHis – Halo – TEV site – CTCF WT – Flag

MGSSHHHHHHSSGTSLYKKAGLMAEIGTGFPFDPHYVEVLGERMHYVDVGPRDGTPVLFLHG
NPTSSYVWRNIIPHVAPTHRCIAPDLIGMGKSDKPDLGYFFDDHVRFMDAFIEALGLEEVVLVIHD
WGSALGFHWAKRNPERVKGIAFMEFIRPIPTWDEWPEFARETFQAFRTTDVGRKLIIDQNVFIEG
TLPMGVVRPLTEVEMDHYREPFLNPVDREPLWRFPNELPIAGEPANIVALVEEYMDWLHQSPV
PKLLFWGTPGVLIPPAEAARLAKSLPNCKAVDIGPGLNLLQEDNPDLIGSEIARWLSTLEISGEPT
TEDLYFQSDNTTLYTKVVMEGDAVEAIVEESETFIKGKERKTYQRRREGGQEEDACHLPQNQTD
GGEVVQDVNSSVQMVMMEQLDPTLLQMKTEVMEGTVAPEAEAAVDDTQIITLQVVNMEEQPINI
GELQLVQVPVPVTVPVATTSVEELQGAYENEVSKEGLAESEPMICHTLPLPEGFQVVKVGANGE
VETLEQGELPPQEDPSWQKDPDYQPPAKKTKKTKKSKLRYTEEGKDVDVSVYDFEEEQQEGLL
SEVNAEKVVGNMKPPKPTKIKKKGVKKTFQCELCSYTCPRRSNLDRHMKSHTDERPHKCHLCG
RAFRTVTLLRNHLNTHTGTRPHKCPDCDMAFVTSGELVRHRRYKHTHEKPFKCSMCDYASVEV
SKLKRHIRSHTGERPFQCSLCSYASRDTYKLKRHMRTHSGEKPYECYICHARFTQSGTMKMHIL
QKHTENVAKFHCPHCDTVIARKSDLGVHLRKQHSYIEQGKKCRYCDAVFHERYALIQHQKSHKN
EKRFKCDQCDYACRQERHMIMHKRTHTGEKPYACSHCDKTFRQKQLLDMHFKRYHDPNFVPA
AFVCSKCGKTFTRRNTMARHADNCAGPDGVEGENGGETKKSKRGRKRKMRSKKEDSSDSEN
AEPDLDDNEDEEEPAVEIEPEPEPQPVTPAPPPAKKRRGRPPGRTNQPKQNQPTAIIQVEDQNT
GAIENIIVEVKKEPDAEPAEGEEEEAQPAATDAPNGDLTPEMILSMMDRDYKDDDDK

CTCF ΔN
6xHis – Halo – TEV site – CTCF (Δ1-265) – Flag

MGSSHHHHHHSSGMAEIGTGFPFDPHYVEVLGERMHYVDVGPRDGTPVLFLHGNPTSSYVWR
NIIPHVAPTHRCIAPDLIGMGKSDKPDLGYFFDDHVRFMDAFIEALGLEEVVLVIHDWGSALGFH
WAKRNPERVKGIAFMEFIRPIPTWDEWPEFARETFQAFRTTDVGRKLIIDQNVFIEGTLPMGVVR
PLTEVEMDHYREPFLNPVDREPLWRFPNELPIAGEPANIVALVEEYMDWLHQSPVPKLLFWGTP
GVLIPPAEAARLAKSLPNCKAVDIGPGLNLLQEDNPDLIGSEIARWLSTLEISGEPTTEDLYFQSG
SFQCELCSYTCPRRSNLDRHMKSHTDERPHKCHLCGRAFRTVTLLRNHLNTHTGTRPHKCPDC
DMAFVTSGELVRHRRYKHTHEKPFKCSMCDYASVEVSKLKRHIRSHTGERPFQCSLCSYASRD
TYKLKRHMRTHSGEKPYECYICHARFTQSGTMKMHILQKHTENVAKFHCPHCDTVIARKSDLGV
HLRKQHSYIEQGKKCRYCDAVFHERYALIQHQKSHKNEKRFKCDQCDYACRQERHMIMHKRTH
TGEKPYACSHCDKTFRQKQLLDMHFKRYHDPNFVPAAFVCSKCGKTFTRRNTMARHADNCAG
PDGVEGENGGETKKSKRGRKRKMRSKKEDSSDSENAEPDLDDNEDEEEPAVEIEPEPEPQPV
TPAPPPAKKRRGRPPGRTNQPKQNQPTAIIQVEDQNTGAIENIIVEVKKEPDAEPAEGEEEEAQP
AATDAPNGDLTPEMILSMMDRDYKDDDDK

CTCF ΔC
6xHis – Halo – TEV site – CTCF (Δ580-727) – Flag

MGSSHHHHHHSSGMAEIGTGFPFDPHYVEVLGERMHYVDVGPRDGTPVLFLHGNPTSSYVWR
NIIPHVAPTHRCIAPDLIGMGKSDKPDLGYFFDDHVRFMDAFIEALGLEEVVLVIHDWGSALGFH
WAKRNPERVKGIAFMEFIRPIPTWDEWPEFARETFQAFRTTDVGRKLIIDQNVFIEGTLPMGVVR
PLTEVEMDHYREPFLNPVDREPLWRFPNELPIAGEPANIVALVEEYMDWLHQSPVPKLLFWGTP
GVLIPPAEAARLAKSLPNCKAVDIGPGLNLLQEDNPDLIGSEIARWLSTLEISGEPTTEDLYFQSD
NTTLYTKVVMEGDAVEAIVEESETFIKGKERKTYQRRREGGQEEDACHLPQNQTDGGEVVQDV
NSSVQMVMMEQLDPTLLQMKTEVMEGTVAPEAEAAVDDTQIITLQVVNMEEQPINIGELQLVQV
PVPVTVPVATTSVEELQGAYENEVSKEGLAESEPMICHTLPLPEGFQVVKVGANGEVETLEQGE

LPPQEDPSWQKDPDYQPPAKKTKKTKKSKLRYTEEGKDVDVSVYDFEEEQQEGLLSEVNAEKV
VGNMKPPKPTKIKKKGVKKTFQCELCSYTCPRRSNLDRHMKSHTDERPHKCHLCGRAFRTVTL
LRNHLNTHTGTRPHKCPDCDMAFVTSGELVRHRRYKHTHEKPFKCSMCDYASVEVSKLKRHIR
SHTGERPFQCSLCSYASRDTYKLKRHMRTHSGEKPYECYICHARFTQSGTMKMHILQKHTENV
AKFHCPHCDTVIARKSDLGVHLRKQHSYIEQGKKCRYCDAVFHERYALIQHQKSHKNEKRFKCD
QCDYACRQERHMIMHKRTHTGEKPYACSHCDKTFRQKQLLDMHFKRYHDPNFVPAAFVCSKC
GKTFTRRNTMARHADNCAGDYKDDDDK

CTCF ΔNC
6xHis – Halo – TEV site – CTCF (Δ1-265; Δ580-727) – Flag

MGSSHHHHHHSSGMAEIGTGFPFDPHYVEVLGERMHYVDVGPRDGTPVLFLHGNPTSSYVWR
NIIPHVAPTHRCIAPDLIGMGKSDKPDLGYFFDDHVRFMDAFIEALGLEEVVLVIHDWGSALGFH
WAKRNPERVKGIAFMEFIRPIPTWDEWPEFARETFQAFRTTDVGRKLIIDQNVFIEGTLPMGVVR
PLTEVEMDHYREPFLNPVDREPLWRFPNELPIAGEPANIVALVEEYMDWLHQSPVPKLLFWGTP
GVLIPPAEAARLAKSLPNCKAVDIGPGLNLLQEDNPDLIGSEIARWLSTLEISGEPTTEDLYFQSG
SFQCELCSYTCPRRSNLDRHMKSHTDERPHKCHLCGRAFRTVTLLRNHLNTHTGTRPHKCPDC
DMAFVTSGELVRHRRYKHTHEKPFKCSMCDYASVEVSKLKRHIRSHTGERPFQCSLCSYASRD
TYKLKRHMRTHSGEKPYECYICHARFTQSGTMKMHILQKHTENVAKFHCPHCDTVIARKSDLGV
HLRKQHSYIEQGKKCRYCDAVFHERYALIQHQKSHKNEKRFKCDQCDYACRQERHMIMHKRTH
TGEKPYACSHCDKTFRQKQLLDMHFKRYHDPNFVPAAFVCSKCGKTFTRRNTMARHADNCAG
DYKDDDDK

CTCF ΔRBR
6xHis – Halo – TEV site – CTCF (Δ264-291; Δ521-614) – Flag

MGSSHHHHHHSSGTSLYKKAGLMAEIGTGFPFDPHYVEVLGERMHYVDVGPRDGTPVLFLHG
NPTSSYVWRNIIPHVAPTHRCIAPDLIGMGKSDKPDLGYFFDDHVRFMDAFIEALGLEEVVLVIHD
WGSALGFHWAKRNPERVKGIAFMEFIRPIPTWDEWPEFARETFQAFRTTDVGRKLIIDQNVFIEG
TLPMGVVRPLTEVEMDHYREPFLNPVDREPLWRFPNELPIAGEPANIVALVEEYMDWLHQSPV
PKLLFWGTPGVLIPPAEAARLAKSLPNCKAVDIGPGLNLLQEDNPDLIGSEIARWLSTLEISGEPT
TEDLYFQSDNTTLYTKVVMEGDAVEAIVEESETFIKGKERKTYQRRREGGQEEDACHLPQNQTD
GGEVVQDVNSSVQMVMMEQLDPTLLQMKTEVMEGTVAPEAEAAVDDTQIITLQVVNMEEQPINI
GELQLVQVPVPVTVPVATTSVEELQGAYENEVSKEGLAESEPMICHTLPLPEGFQVVKVGANGE
VETLEQGELPPQEDPSWQKDPDYQPPAKKTKKTKKSKLRYTEEGKDVDVSVYDFEEEQQEGLL
SEVNAEKVVGNMKPPKPTKIKKKGVKRPHKCHLCGRAFRTVTLLRNHLNTHTGTRPHKCPDCD
MAFVTSGELVRHRRYKHTHEKPFKCSMCDYASVEVSKLKRHIRSHTGERPFQCSLCSYASRDT
YKLKRHMRTHSGEKPYECYICHARFTQSGTMKMHILQKHTENVAKFHCPHCDTVIARKSDLGVH
LRKQHSYIEQGKKCRYCDAVFHERYALIQHQKSHKNEKRFKCDQCDYACRQERHMIMHKRTHT
GEAEPDLDDNEDEEEPAVEIEPEPEPQPVTPAPPPAKKRRGRPPGRTNQPKQNQPTAIIQVEDQ
NTGAIENIIVEVKKEPDAEPAEGEEEEAQPAATDAPNGDLTPEMILSMMDRDYKDDDDK

ZF4-7
6xHis – CTCF (Δ1-350; Δ461-727) – Flag

MGSSHHHHHHSSGFKCSMCDYASVEVSKLKRHIRSHTGERPFQCSLCSYASRDTYKLKRHMR
THSGEKPYECYICHARFTQSGTMKMHILQKHTENVAKFHCPHCDTVIARKSDLGVHLRKQHDYK
DDDDK

ZF9-CT
6xHis – CTCF (Δ1-489) – Flag

MGSSHHHHHHSSGLVPRGSHMKNEKRFKCDQCDYACRQERHMIMHKRTHTGEKPYACSHCD
KTFRQKQLLDMHFKRYHDPNFVPAAFVCSKCGKTFTRRNTMARHADNCAGPDGVEGENGGET
KKSKRGRKRKMRSKKEDSSDSENAEPDLDDNEDEEEPAVEIEPEPEPQPVTPAPPPAKKRRGR
PPGRTNQPKQNQPTAIIQVEDQNTGAIENIIVEVKKEPDAEPAEGEEEEAQPAATDAPNGDLTPE
MILSMMDRDYKDDDDK

SA1
10xHis – SA1 WT – S6

MHHHHHHHHHHSGGSMITSELPVLQDSTNETTAHSDAGSELEETEVKGKRKRGRPGRPPSTN
KKPRKSPGEKSRIEAGIRGAGRGRANGHPQQNGEGEPVTLFEVVKLGKSAMQSVVDDWIESYK
QDRDIALLDLINFFIQCSGCRGTVRIEMFRNMQNAEIIRKMTEEFDEDSGDYPLTMPGPQWKKFR
SNFCEFIGVLIRQCQYSIIYDEYMMDTVISLLTGLSDSQVRAFRHTSTLAAMKLMTALVNVALNLSI
HQDNTQRQYEAERNKMIGKRANERLELLLQKRKELQENQDEIENMMNSIFKGIFVHRYRDAIAEI
RAICIEEIGVWMKMYSDAFLNDSYLKYVGWTLHDRQGEVRLKCLKALQSLYTNRELFPKLELFTN
RFKDRIVSMTLDKEYDVAVEAIRLVTLILHGSEEALSNEDCENVYHLVYSAHRPVAVAAGEFLHK
KLFSRHDPQAEEALAKRRGRNSPNGNLIRMLVLFFLESELHEHAAYLVDSLWESSQELLKDWEC
MTELLLEEPVQGEEAMSDRQESALIELMVCTIRQAAEAHPPVGRGTGKRVLTAKERKTQIDDRN
KLTEHFIITLPMLLSKYSADAEKVANLLQIPQYFDLEIYSTGRMEKHLDALLKQIKFVVEKHVESDV
LEACSKTYSILCSEEYTIQNRVDIARSQLIDEFVDRFNHSVEDLLQEGEEADDDDIYNVLSTLKRL
TSFHNAHDLTKWDLFGNCYRLLKTGIEHGAMPEQIVVQALQCSHYSILWQLVKITDGSPSKEDLL
VLRKTVKSFLAVCQQCLSNVNTPVKEQAFMLLCDLLMIFSHQLMTGGREGLQPLVFNPDTGLQS
ELLSFVMDHVFIDQDEENQSMEGDEEDEANKIEALHKRRNLLAAFSKLIIYDIVDMHAAADIFKHY
MKYYNDYGDIIKETLSKTRQIDKIQCAKTLILSLQQLFNELVQEQGPNLDRTSAHVSGIKELARRF
ALTFGLDQIKTREAVATLHKDGIEFAFKYQNQKGQEYPPPNLAFLEVLSEFSSKLLRQDKKTVHS
YLEKFLTEQMMERREDVWLPLISYRNSLVTGGEDDRMSVNSGSSSSKTSSVRNKKGRPPLHKK
RVEDESLDNTWLNRTDTMIQTPGPLPAPQLTSTVLRENSRPMGDQIQEPESEHGSEPDFLHNP
QMQISWLGQPKLEDLNRKDRTGMNYMKVRTGVRHAVRGLMEEDAEPIFEDVMMSSRSQLEDM
NEEFEDTMVIDLPPSRNRRERAELRPDFFDSAAIIEDDSGFGMPMFGSGSGGMGDSLSWLLRLL
N

SA2
10xHis – SA2 WT – ybbR

MHHHHHHHHHHGSGSSGIAAPEIPTDFNLLQESETHFSSDTDFEDIEGKNQKQGKGKTCKKGK
KGPAEKGKGGNGGGKPPSGPNRMNGHHQQNGVENMMLFEVVKMGKSAMQSVVDDWIESYK
HDRDIALLDLINFFIQCSGCKGVVTAEMFRHMQNSEIIRKMTEEFDEDSGDYPLTMAGPQWKKF
KSSFCEFIGVLVRQCQYSIIYDEYMMDTVISLLTGLSDSQVRAFRHTSTLAAMKLMTALVNVALNL
SINMDNTQRQYEAERNKMIGKRANERLELLLQKRKELQENQDEIENMMNAIFKGVFVHRYRDAI
AEIRAICIEEIGIWMKMYSDAFLNDSYLKYVGWTMHDKQGEVRLKCLTALQGLYYNKELNSKLEL
FTSRFKDRIVSMTLDKEYDVAVQAIKLLTLVLQSSEEVLTAEDCENVYHLVYSAHRPVAVAAGEF
LYKKLFSRRDPEEDGMMKRRGRQGPNANLVKTLVFFFLESELHEHAAYLVDSMWDCATELLKD
WECMNSLLLEEPLSGEEALTDRQESALIEIMLCTIRQAAECHPPVGRGTGKRVLTAKEKKTQLD
DRTKITELFAVALPQLLAKYSVDAEKVTNLLQLPQYFDLEIYTTGRLEKHLDALLRQIRNIVEKHTD
TDVLEACSKTYHALCNEEFTIFNRVDISRSQLIDELADKFNRLLEDFLQEGEEPDEDDAYQVLSTL
KRITAFHNAHDLSKWDLFACNYKLLKTGIENGDMPEQIVIHALQCTHYVILWQLAKITESSSTKED
LLRLKKQMRVFCQICQHYLTNVNTTVKEQAFTILCDILMIFSHQIMSGGRDMLEPLVYTPDSSLQS
ELLSFILDHVFIEQDDDNNSADGQQEDEASKIEALHKRRNLLAAFCKLIVYTVVEMNTAADIFKQY
MKYYNDYGDIIKETMSKTRQIDKIQCAKTLILSLQQLFNEMIQENGYNFDRSSSTFSGIKELARRF
ALTFGLDQLKTREAIAMLHKDGIEFAFKEPNPQGESHPPLNLAFLDILSEFSSKLLRQDKRTVYVY

LEKFMTFQMSLRREDVWLPLMSYRNSLLAGGDDDTMSVISGISSRGSTVRSKKSKPSTGKRKV
VEGMQLSLTEESSSSDSMWLSREQTLHTPVMMQTPQLTSTIMREPKRLRPEDSFMSVYPMQT
EHHQTPLDYNRRGTSLMEDDEEPIVEDVMMSSEGRIEDLNEGMDFDTMDIDLPPSKNRRERTE
LKPDFFDPASIMDESVLGVSMFGSSGDSLEFIASKLA

Rad21
6x His – Rad21 (Δ1-280; Δ421-631) – TEV site – MBPx

MGSSHHHHHHSGVDPVEPMPTMTDQTTLVPNEEEAFALEPIDITVKETKAKRKRKLIVDSVKEL
DSKTIRAQLSDYSDIVTTLDLAPPTKKLMMWKETGGVEKLFSLPAQPLWNNRLLKLFTRCLTPLV
PEDLRKRRKGGEADNLDEFLKEFGSSGENLYFQGGSGSNSSSGGSGGGSGKIEEGKLVIWING
DKGYNGLAEVGKKFEKDTGIKVTVEHPDKLEEKFPQVAATGDGPDIIFWAHDRFGGYAQSGLLA
EITPDKAFQDKLYPFTWDAVRYNGKLIAYPIAVEALSLIYNKDLLPNPPKTWEEIPALDKELKAKG
KSALMFNLQEPYFTWPLIAADGGYAFKYGDIKDVGVDNAGAKAGLTFLVDLIKNKHMNADTDYSI
AEAAFNKGETAMTINGPWAWSNIDTSKVNYGVTVLPTFKGQPSKPFVGVLSAGINAASPNKELA
KEFLENYLLTDEGLEAVNKDKPLGAVALKSYEEELVKDPRVAATMENAQKGEIMPNIPQMSAFW
YAVRTAVINAASGRQTVDEALKDAQT
